# Research progress of non-coding RNA in atrial fibrillation

**DOI:** 10.3389/fcvm.2023.1210762

**Published:** 2023-07-14

**Authors:** Zongqian Xue, Jinbiao Zhu, Juan Liu, Lingli Wang, Jijun Ding

**Affiliations:** Department of Cardiology, Aoyang Hospital Affiliated to Jiangsu University, Zhangjiagang, China

**Keywords:** atrial fibrillation, ncRNAs, biomarker, diagnosis, exosome

## Abstract

Atrial fibrillation (AF) is a common arrhythmia in clinic, and its incidence is increasing year by year. In today's increasingly prevalent society, ageing poses a huge challenge to global healthcare systems. AF not only affects patients' quality of life, but also causes thrombosis, heart failure and other complications in severe cases. Although there are some measures for the diagnosis and treatment of AF, specific serum markers and targeted therapy are still lacking. In recent years, ncRNAs have become a hot topic in cardiovascular disease research. These ncRNAs are not only involved in the occurrence and development of AF, but also in pathophysiological processes such as myocardial infarction and atherosclerosis, and are potential biomarkers of cardiovascular diseases. We believe that the understanding of the pathophysiological mechanism of AF and the study of diagnosis and treatment targets can form a more systematic diagnosis and treatment framework of AF and provide convenience for individuals with AF and the society.

## 1. Introduction

Atrial fibrillation (AF) is a common arrhythmias in clinic, with a high risk of death, stroke, and peripheral embolism, and its incidence has been increasing year by year. Risk factors for AF are closely related to cardiovascular disease, with organic or functional heart problems being more common. In addition, age, gender and genetic factors are also important factors leading to the occurrence of AF (1, 2). AF not only affects life quality of the patients, but also has complications such as thrombosis and heart failure in severe cases. Atrial remodeling is considered to be the basis of the occurrence and development of AF, including structural remodeling, electrical remodeling, neural remodeling, etc (3–5). The diagnosis of AF mainly depends on electrocardiogram findings, which are often found after complications occur, and there is a certain lag (6). Therefore, biomarkers have potential value in the early diagnosis of AF. Currently, drug therapy for AF patients has poor efficacy and side effects. Radiofrequency ablation is more effective than drug therapy, but the patients are yet able to avoid the operational risks, postoperative recurrence, and high healthcare cost (7–9). Actively searching for new diagnosis and treatment strategies and exploring the molecular mechanism of AF have great clinical significance and translational prospects.

**Figure 1:**
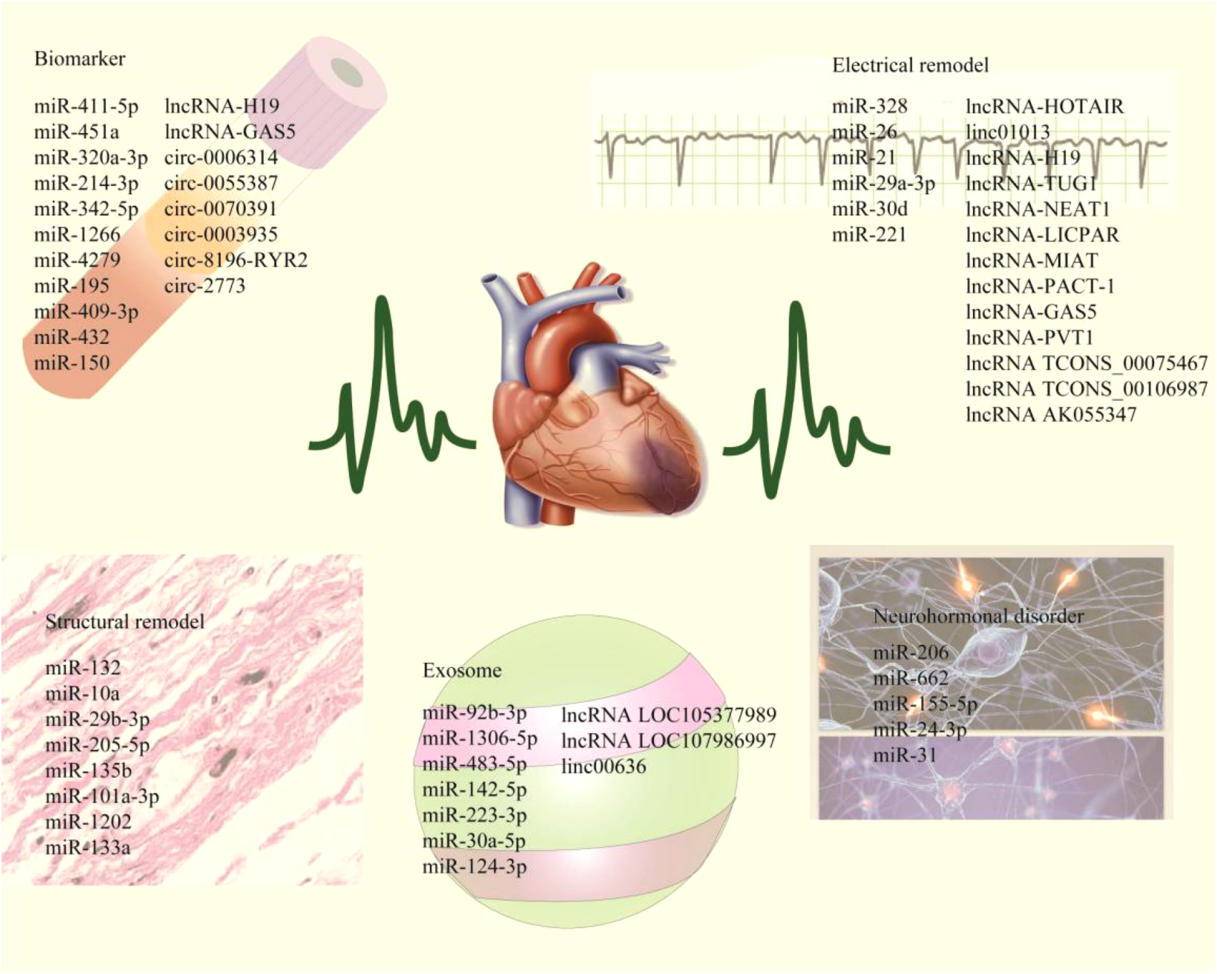
The schematic highlights ncRNAs associated with myocardial electrical remodeling, fibrosis, neurohormone disorders, and exosomes, while including plasma markers associated with AF diagnosis and prognotic monitoring.

In recent years, non-coding RNA (ncRNA) has become a research hotspot in cardiovascular diseases. ncRNA mainly includes miRNA, LncRNA and CircRNA. These ncRNAs can not only participate in the occurrence and development of AF, but also play a part in the pathophysiological processes such as myocardial infarction and atherosclerosis, which are potential biomarkers for cardiovascular diseases (10). This article reviews the pathophysiological mechanism of AF, introduces the mechanism and potential value of ncRNAs in AF, and provides a theoretical basis for the diagnosis, treatment and prognosis monitoring of AF (Figure 1, Tables 1, 2).

**Table 1:** ncRNAs involved in the pathophysiology of AF.

ncRNAs	Expression	Remodeling	Targets	Ref.
miR-205-5p	Downregulation	Structural remodeling	EHMT2/IGFBP3	(11)
miR-181b	Upregulation	Structural remodeling	Sema3A	(12)
miR-423	Downregulation	Electrical remodeling	Calcium handling protein	(13)
miR-29b	Downregulation	Structural remodeling	TGFβR1	(14)
miR-34a	Upregulation	Electrical remodeling	TASK1	(15)
miR-21	Upregulation	Structural remodeling	IL-18 FGFR1	(16)
miR-662	Upregulation	Electrical remodeling Neurohormonal disorders	CREB1	(17)
miR-425-5p	Upregulation	Structural remodeling	CREB1	(18)
miR-135b	Downregulation	Structural remodeling	TGFβR1	(19)
miR-146b-5p	Upregulation	Structural remodeling	TIMP4	(20)
miR-199a-5p	Upregulation	Electrical remodeling	NCX	(21)
miR-22-5p	Upregulation	Electrical remodeling	NCX	(21)
miR-101a-3p	Downregulation	Structural remodeling	EZH2	(22)
miR-1202	Upregulation	Structural remodeling	nNOS TGFβ1	(23)
miR-133a	Downregulation	Structural remodeling	CTGF	(24)
miR-205	Upregulation	Structural remodeling	P4HA3	(11, 25)
miR-4443	Upregulation	Structural remodeling	THBS1	(26)
miR-155	Upregulation	Electrical remodeling	CACNA1C	(27)
miR-29b-3p	Downregulation	Structural remodeling	PDGFB	(28, 29)
miR-324-3p	Downregulation	Structural remodeling	TGFβ1	(30)
miR-210	Upregulation	Structural remodeling	Foxp3	(31)
miR-27b-3p	Downregulation	Structural remodeling	CX43	(32)
miR-23	Upregulation	Structural remodeling	TGFβ1	(33)
miR-133	Downregulation	Structural remodeling	ZFHX3	(34)
miR-10a	Upregulation	Structural remodeling	TGFβ1 Smads	(35)
miR-155-5p	Upregulation	Neurohormonal disorders	eNOS	(36)
miR-24-3p	Upregulation	Neurohormonal disorders	eNOS	(36)
miR-138-5p	Downregulation	Structural remodeling	CYP11B2	(37)
miR-27b	Downregulation	Structural remodeling	ALK5	(32, 38)
miR-30c	Downregulation	Structural remodeling	TGFβRII	(39)
miR-208b	Upregulation	Electrical remodeling	CACNA1C CACNB2 SERCA2	(40)
miR-29a	Upregulation	Electrical remodeling	CACNA1C	(41)
miR-31	Upregulation	Neurohormonal disorders	nNOS	(42)
miR-30d	Upregulation	Electrical remodeling	IK.ACh	(43)
miR-30a	Upregulation	Structural remodeling	Snail 1	(44)
miR-206	Upregulation	Neurohormonal disorders	SOD1	(45)
miR-146b-5p	Upregulation	Structural remodeling	TIMP4	(20)
miR-132	Downregulation	Structural remodeling	CTGF	(46)
miR-106b-25	Downregulation	Electrical remodeling	RyR2	(47)
miR-21	Upregulation	Electrical remodeling	CACNA1C CACNB2	(16, 48)
miR-26	Downregulation	Electrical remodeling	KCNJ2	(49)
miR-221	Upregulation	Electrical remodeling	KCNJ5	(50)
miR-499	Upregulation	Electrical remodeling	SK3	(51, 52)
miR-328	Upregulation	Electrical remodeling	CACNA1C CACNB2	(51)
HOTAIR	Upregulation	Structural remodeling	PTBP1 Wnt5a	(9)
H19	Upregulation	Structural remodeling	VEGFA TGFβ	(53)
NEAT1	Upregulation	Structural remodeling	NPAS2	(54)
LICPAR	Upregulation	Structural remodeling	Smad2/3	(55)
LINC01013	Upregulation	Structural remodeling	TGF-β1	(56)
TUG1	Upregulation	Structural remodeling	miR-29b-3p	(57)
PCAT-1	Upregulation	Structural remodeling	TGF-β1	(58)
TCONS00106987	Upregulation	Electrical remodeling	KCNJ2	(59)
GAS5	Upregulation	Structural remodeling	ALK5	(60)
MIAT	Upregulation	Structural remodeling	TGFβ1	(61)
PVT1	Upregulation	Structural remodeling	TGFβ1	(62)
KCNQ1OT1	Upregulation	Electrical remodeling	CACNA1C	(63)
AK055347	Upregulation	Neurohormonal disorders	MSS51	(64)
CAMTA1	Upregulation	Structural remodeling	TGFBR1	(65)
circ_0004104	Upregulation	Structural remodeling	TGFβ	(66)
circ_0000672	Upregulation	Structural remodeling	TRAF6	(67)
circ_0005019	Upregulation	Electrical remodeling	Kcnn3	(68)

**Table 2:** Potential biomarker of ncRNAs for AF.

ncRNAs	Expression	Biological fluid	Function	Ref
miR-411-5p	Upregulation	Blood	Auxiliary diagnostic	(69)
miR-451a	Downregulation	Blood	Prognostic monitor	(70)
miR-320a-3p	Upregulation	Plasma	Prognostic monitor	(28)
miR-214-3p	Upregulation	Serum	Auxiliary diagnostic	(71)
miR-342-5p	Upregulation	Serum	Auxiliary diagnostic	(71)
miR-1266	Upregulation	Bloo	Auxiliary diagnostic	(72)
miR-4279	Upregulation	Blood	Auxiliary diagnostic	(72)
miR-4666a-3p	Upregulation	Blood	Auxiliary diagnostic	(72)
miR-208a	Downregulation	Serum	Auxiliary diagnostic	(73)
miR-483-5p	Upregulation	Serum	Prognostic monitor	(73)
miR-199a	Downregulation	Blood	Prognostic monitor	(74)
miR-409-3p	Downregulation	Plasma	Prognostic monitor	(75)
miR-432	Downregulation	Plasma	Prognostic monitor	(75)
miRNA-150	Downregulation	Plasma	Auxiliary diagnostic	(76)
lncRNA H19	Upregulation	Plasma	Prognostic monitor	(77)
LncRNA GAS5	Downregulation	Plasma	Auxiliary diagnostic Prognostic monitor	(78)
has_circ_0006314	Upregulation	Blood	Prognostic monitor	(79)
hsa_circ_0055387	Upregulation	Blood	Prognostic monitor	(79)
hsa_circ_0070391	Upregulation	Plasma	Auxiliary diagnostic	(80)
hsa_circ_0003935	Downregulation	Plasma	Auxiliary diagnostic	(80)
circ 8196-RYR2	Upregulation	Blood	Prognostic monitor	(81)
circRNA_2773	Upregulation	PBMC	Auxiliary diagnostic	(82)

## 2. Pathophysiology of AF

AF can be classified as paroxysmal, persistent and permanent according to the duration of the attack. The pathogenesis of AF involves a variety of factors, mainly including electrical remodeling, structural remodeling, and neurohormonal disorders. These mechanisms lead to the development and maintenance of AF (83, 84). Although the pathogenesis of AF is complex, it is mainly related to electrical remodeling and structural remodeling. Existing studies suggest that ncRNAs play an important role in its occurrence and development (Table 1) (85–87).

## 3. miRNAs involved in the diagnosis and prognostic monitoring of AF

The early symptoms of AF are not obvious, the main clinical manifestations are palpitation, dyspnea and dizziness, which are easy to be ignored by patients, and routine electrocardiogram is difficult to monitor, so the diagnosis is often missed (88, 89). At present, BNP and troponin are the main clinical biomarkers for the diagnosis of cardiovascular diseases, but they are mainly used for the diagnosis of heart failure and myocardial infarction, and have no significant significance for the diagnosis of AF. In recent years, the research on ncRNA has become increasingly in-depth. The differential expression of ncRNAs in cardiac tissue and blood of patients with AF may become auxiliary diagnostic biomarkers for AF (Table 2) (90, 91).

Risk stratification of subsequent cardiovascular events in patients with AF helps guide prevention strategies. Nossent AY et al. analyzed differentially expressed miRNAs in 26 patients using sequencing technology, and screened out one miR-411-5p in combination with clinical prognosis as a potential valuable prognostic biomarker for patients with AF (69). Recurrent AF after catheter ablation seriously affected the prognosis of patients. Therefore, Garcia-Seara J et al. recruited 42 patients with AF for catheter ablation. The analysis measured the expression of 84 miRNAs in both non-relapsed and relapsed groups, the results showed that miRNA-451a was down-regulated in relapsed patients, and the recurrence of AF was positively correlated with an increased percentage of scars. It is suggested that low expression of miR-451a may play an important role in the recurrence of AF by controlling fibrosis and progression (70). Akselrod AS et al. found that plasma miR-320a-3p level in patients with AF was higher than that in healthy controls, and the expression level was positively correlated with CHADS-VASc score (28). Sasano T et al. identified 11 candidate miRNAs using high-throughput sequencing and clinical sample validation, and found that miR-214-3p and miR-342-5p had high accuracy in the diagnosis of patients with AF combined with clinicpathological parameter analysis (71). Yang et al. observed genome-wide differential expression profiles of miRNAs in 180 peripheral blood samples and found 14 miRNAs with significant differential expression, among which miR-1266, miR-4279 and miR-4666a-3p were significantly increased in expression, which are potential targets for future diagnosis and treatment of AF (72). About one-third of patients undergoing coronary artery bypass grafting will develop postoperative AF, which seriously affects the prognosis of patients. In order to monitor the occurrence of postoperative AF, Athanasiou et al. prospectively recruited 34 patients after surgery, and compared the myocardial tissue with normal sinus rhythm after surgery, and found 16 differentially expressed miRNAs. The expression of miR-208a was significantly decreased, and the expression of miR-483-5p was significantly increased. It is suggested that these differentially expressed miRNAs can be used to predict the recurrence of AF after coronary artery bypass grafting (73). Kilic et al. recruited 63 patients after coronary artery bypass grafting and monitored their heart rate until discharge. Among them, 20 patients developed postoperative AF, and PCR detected the expression of miR-199a and miR-195. The results showed that the expression of miR-199a significantly decreased in the postoperative AF group, demonstrate its effectiveness as a biomarker for cardiac surgery management (74). By Solexa sequencing 100 patients with AF who underwent catheter ablation and 100 healthy individuals, Wu et al. found that miR-409-3p and miR-432 were significantly reduced in the plasma of patients with AF and are potential markers of AF (75). Xia et al. showed for the first time that plasma miRNA-150 levels in patients with atrial fibrillation are significantly lower than those in healthy individuals, which is a potential biomarker to aid in the diagnosis of atrial fibrillation (76). These studies indicate that miRNAs differentially expressed in plasma of patients with AF and postoperative patients can play an important indicator role in the diagnosis and prognosis monitoring of AF.

## 4. miRNAs involved in the regulation of electrical remodeling

Electrical remodeling of atrial muscle is closely related to the occurrence of AF. Electrical remodeling refers to recurrent episodes of AF or continuous atrial stimulation, which leads to progressive shortening of the effective refractory period of the atrium, and the decrease, reversal or disappearance of the physiological frequency adaptation of the atrial refractory period, making AF more likely to be induced and sustained (87, 92). AF is caused by abnormal electrical activity of atrial myocardium. During the occurrence of AF, many ion channels also have significant changes, mainly including: L-type Ca^2+^ channel, transient outward K^+^ channel, strong inward rectification K^+^ channel (IK1), acetylcholine-activated K^+^ channel (IK, ACh), and ultra-fast delayed rectification K^+^ channel (IKur) (93, 94).

Yang et al. found that the expression of miR-328 was increased in the atrial tissue of AF mouse models, and the high expression of miR-328 could reduce the L-type Ca^2+^ current and shorten the duration of atrial action potential. Mechanism studies have confirmed that CACNA1C and CACNB1 are the target genes of miR-328, and miR-328 can interact with L-type Ca^2+^ channel protein subunits to participate in atrial electroremodeling in AF (51). Nattel et al. found that the expression of miR-26 was down-regulated in the atrial tissues of AF patients, and low-expressed miR-26 was a potential regulatory gene for the electrophysiological effects of Ca^2+^ dependent nuclear factor of activated T cells (NFAT) signaling pathway, and an important participant in the persistence of AF (49). Ricardo et al. found that the high expression of miR-21 in cardiomyocytes of patients with AF was negatively correlated with the expression of CACNA1C and the density of I (Ca, L), suggesting that miR-21 may be involved in the downregulation of L-type Ca^2+^ I (Ca, L) induced by chronic AF, and is the key to the persistence of AF (95). Similarly, Qiu et al. found that CACNA1C is a direct target gene of miR-29a-3p, and miR-29a-3p negatively regulates CACNA1C. miR-29a-3p may be a potential target for AF treatment (41). Lee et al. found that miR-499 was significantly upregulated in AF, resulting in downregulation of small conductance calcium-activated potassium channel 3 (SK3), which may contribute to electrical remodeling of AF and is a novel site associated with the onset of AF (52). Katsushige et al. used high-throughput sequencing analysis to find that miR-30d was significantly up-regulated in myocardial cells of AF patients, and functional enrichment analysis found that miR-30d was a candidate gene for ion channel remodeling. Interference with miR-30d down-regulated the expression of kcnj3/Kir3.1, accompanied by a decrease in the acetylcholine-sensitive internal rectification K^+^ current (IK.ACh) (43). Barbara et al. found that miR-221 reduced the abundance and function of L-type Ca^2+^ channels and Kcnj5 channels. MiR-221 can regulate L-type Ca^2+^ channels and Kcnj5 channels, thus potentially contributing to the generation and propagation of cardiac excitation (50).

## 5. miRNAs involved in the regulation of structural remodeling

Electrical remodeling is the pathological change in the initial stage of AF, while structural remodeling is the material basis for the long-term maintenance of AF, and it is also the most obvious change of atrium (96, 97). Atrial dilatation and fibrosis are the main features of structural remodeling in AF. Atrial fibrosis may lead to slowing of conduction velocity, conduction block to promote reentry and increase susceptibility to AF (98).

Studies have shown that connective tissue growth factor (CTGF) plays an important role in the process of fibrosis. Zhang et al. found that the expression of miR-132 decreased in AF cardiomyocytes. Luciferase assay confirmed that miR-132 could bind to the 3 ‘-untranslated region of CTGF, thereby inhibiting the expression of CTGF and regulating the fibrosis of cardiac fibroblasts (46). Yang et al. found that overexpression of miR-10a significantly prolonged the duration of AF and decreased Smad7 protein expression. TGF-β1 reversed the inhibitory effect of miR-10a on Smad7, alleviated atrial remodeling, and ultimately inhibited cardiac fibrosis (35). Similarly, Xu et al. found that miR-29b-3p could reduce the degree of atrial fibrosis, and high expression of miR-29b-3p could reduce the expression of fibrosis markers collagen- I and a-SMA, and increase the protein expression of Cx43, thus reversing atrial remodeling (29). Studies have shown that the expression of miR-205-5p is decreased in atrial tissues of patients with AF, and overexpression of miR-205-5p can reduce the expression of TGF-β1, α-SMA, Col III and other fibrosis-related proteins. Mechanism studies have shown that miR-205-5p regulates H3 histone methylation by targeting EHMT2, promotes IGFBP3 expression, and further affects atrial myocyte fibrosis (11). The study found that the expression of miR-29b was low in the atrial tissue of AF rats, overexpression miR-29b can reduce atrial fibrosis, reduce the expression of COL1A1, COL3A1 and TGFβ1, and shorten the duration of AF in rats (14). In addition, the expression of miR-135b was down-regulated in AF tissues, while the expression of miR-135b target genes TGFBR1 and TGFBR2 was up-regulated in myocardial fibroblasts. Quercetin can promote miR-135b expression, inhibit TGF-β/Smads pathway, reduce atrial tissue fibrosis and collagen deposition, and thus relieve AF (19). Xu et al. found that miR-101a-3p may prevent AF in rats by targeting EZH2 to inhibit collagen synthesis and atrial fibrosis, which provides a potential target for the prevention of AF (22). miR-1202 was found to negatively regulate atrial fibrosis by targeting nNOS by reducing cell differentiation, collagen deposition, and TGF-β1/Smad2/3 pathway activity (23). Overexpression of miR-133a can inhibit the proliferation and migration of atrial cells, reduce the expression of fibrosis markers and CTGF protein, and improve myocardial fibrosis (24).

## 6. miRNAs involved in the regulation of neurohormonal disorders

Autonomic dysfunction is a type of dysfunction that occurs when the balance between sympathetic and parasympathetic nerves is disrupted. cardiac autonomic nerve remodeling (ANR) refers to the changes in the distribution density and spatial arrangement of the autonomic nerve caused by some diseases of the heart (99–102).

Studies have shown that the contents of tetrahydrobioterin (BH4) and NO are related to nerve regeneration. GCH1 is the rate-limiting enzyme of BH4 synthesis. Hou et al. found that the expression of miR-206 was increased in atrial fibrillation myocarde. High expression of miR-206 could inhibit GCH1, thus affecting the content of BH4 and NO in myocarde (103). In a similar study, miR-206 expression was increased in the the left superior ganglionated plexus (SLGPs). High expression of miR-206 inhibited the expression of superoxide dismutase 1 (SOD1) and increased the levels of reactive oxygen species (ROS) *in vitro* and *in vivo*, further exacerbating ANR (45). miR-662 can also regulate the expression of neuropeptides and participate in the occurrence and development of AF after myocardial infarction (17). It was found that the levels of miR-155-5p and miR-24-3p were significantly decreased and the levels of eNOS and NO were increased in patients with AF after ablation compared with those who did not receive ablation therapy (36). Casadei B et al. found that atrial specific upregulation of miR-31 in AF resulted in inhibition of muscular dystrophin (DYS) translation and accelerated degradation of nNOS mRNA, leading to significant reductions in atrial DYS and nNOS protein content and nitric oxide availability. Inhibition of miR-31 restores DYS and nNOS in human AF and normalizes APD and rate dependence of APD (42).

## 7. ncRNAs and AF-beyond miRNAs

With the increase of studies on ncRNAs in AF, lncRNAs and circRNAs play an increasingly significant role in AF. Therefore, in addition to miRNAs, this manuscript also discussed the current research content of other ncRNAs in AF.

CHA 2 ds2-VASc score was originally used to stratify stroke risk in patients with AF, in order to study whether lncRNAs could improve the predictive ability of CHA 2 ds2-VASc score for stroke. Li et al. added the ability of lncRNA expression level to predict stroke in CHA 2 ds2-VASc scoring model. The results showed that lncRNA H19 plasma expression level was correlated with the risk of stroke in patients with AF, which could significantly improve the ability to predict the risk of stroke in patients with AF, and was a potential prognostic monitoring marker (77). LncRNA GAS5 is significantly down-regulated in the plasma of patients with AF, which is a potential biomarker for the diagnosis and prognosis monitoring of AF (78). Similar studies have found that has_circ_0006314 and hsa_circ_0055387 also have potential predictive value for postoperative AF (79). Fan et al. used GEO database to screen out two different circRNAs. The expression of hsa_circ_0070391 in plasma was up-regulated and hsa_circ_0003935 down-regulated. The area under ROC curve indicated that both of them had high diagnostic efficiency (80). Wang et al. examined plasma circ 8196-RYR2 levels in 136 patients following ablation of AF, suggesting that circ 8196-RYR2 could be used as a new predictor of late recurrence after surgical ablation (81). Another study also show that low expression of circRNA_2773 is a potential diagnostic marker for AF (82).

AF is often accompanied by excessive proliferation of cardiac fibroblasts (CFs). It was found that the expression of HOTAIR was increased in the myocardium of patients with AF, and Ang II significantly increased the activity of atrial fibroblasts. HOTAIR knockdown can significantly inhibit AF cardiac tissue fibrosis by regulating Wnt signaling pathway (9). Knocking down LINC01013 reduced baseline expression of fibrosis markers and their response to TGF-β1. TGF-β1 stimulated atrial fibroblasts to induce the expression of LINC01013, and its knockdown reduced the activation of fibroblasts (56). Plasma H19 levels were significantly higher in patients with AF compared with healthy volunteers. Upregulation of H19 expression contributes to the proliferation and synthesis of extracellular matrix (ECM) related proteins, thereby promoting myocardial fibrosis (53). It was found that the serum TUG1 level was elevated and the expression of miR-29b-3p was low in patients with AF. Pearson correlation analysis showed that TUG1 was negatively correlated with miR-29b-3p expression in AF patients. TUG1 knockdown inhibits vascular endothelium-induced cardiomyocyte proliferation (57). NEAT1 expression was up-regulated in atrial tissues of patients with AF, and was positively correlated with the expression of type I collagen (coll I) and type III collagen (coll III). In addition, the loss of NEAT1 attenuates angiotensin II (Ang II), leading to atrial fibroblast proliferation, migration, and collagen production. These findings suggest that NEAT1 plays an important role in atrial fibrosis and is a new potential molecular target for the treatment of AF (54). In AF patients, LICPAR and TGF-β1 expression were up-regulated and positively correlated. Further analysis showed that Ang II increased LIPCAR, Smad2/3 phosphorylation, and α-smooth muscle actin (α-SMA) levels. Up-regulation of LIPCAR could further promote the promoting effects of Ang II on the phosphorylation levels of LIPCAR, Collagen I, Collagen II, α-SMA and Smad2/3, cell viability and proliferation of atrial fibroblasts. These studies suggest that lncRNA LICPAR regulates atrial fibrosis primarily by regulating the TGF-β/Smad pathway (55). Studies found that down-regulation of lncRNA MIAT could significantly relieve AF, increase atrial effective refractory period (AERP), inhibit the expression of fibrosis-related genes coll I, coll III, CTGF, TGF-β1, and effectively reduce AF induced atrial fibrosis (61). PCAT-1 expression was increased in AF patients. PCAT-1 knockdown inhibited the proliferation of AC16 cells. Mechanism studies showed that TGF-β1 was the target of PCAT-1, and its expression in AF tissues was positively correlated with that of PCAT-1. PCAT-1 can promote the proliferation of AF cells by promoting TGF-β1 (58). The expression of GAS5 in myocardium of AF patients was significantly decreased. Overexpression of GAS5 can inhibit the growth of AC16 cells. In addition, further experiments showed that ALK5 was the target of GAS5, and its expression in AF tissue was negatively correlated with that of GAS5. lncRNA GAS5 may inhibit AF cell fibrosis by inhibiting ALK5 (60). The expression of PVT1 in AF patients was increased and positive for coll I and coll III. Overexpression of PVT1 promoted Ang-II-induced atrial fibroblast proliferation, collagen generation, and TGF-β1/Smad signaling activation, while PVT1 knockdown did the opposite. Mechanically, PVT1 acts as a sponge for miR-128-3p and promotes Sp1 expression, thereby activating the TGF-β1/Smad signaling pathway (62).

Hou et al. found that lncRNA TCONS_00075467 may also participate in atrial myocardial electrical remodeling. Interference with TCONS_00075467 can shorten the effective refractory period of the atria *in vivo* and reduce the duration of L-type calcium current and action potential *in vitro* (104). Similarly, lncRNA TCONS-00106987 is up-regulated in atrial tissue of patients with AF. Mechanism studies have shown that TCONS_00106987 induces the transcription of its target gene KCNJ2 through miR-26, and increases the inward rectification K^+^ current (IK1). Thus facilitating electrical reconfiguration (59). Studies have shown that interference with lncRNA AK055347 can inhibit the activity of cardiomyocytes, accompanied by the downregulation of Cyp450 and ATP synthase. Mechanism studies have confirmed that AK055347 may regulate the mitochondrial energy production by regulating Cyp450, ATP synthase and MSS51, thus participating in the pathogenesis of AF (64).

## 8. Exosome-associated ncRNAs involved in the regulation of AF

In recent years, it has been found that exosome-derived ncRNAs have different expression profiles in various diseases and are a potential non-invasive diagnostic biomarker, which has been widely studied in the medical field. Similarly, exosomes can also be detected in body fluids of patients with atrial fibrillation, and the non-coding RNA carried by them is of great significance for auxiliary diagnosis and prognostic monitoring of AF (105, 106).

Wei et al. demonstrated differences in the expression of miRNAs in plasma exosomes in patients with AF. Among them, miR-92b-3p, miR-1306-5p and miR-let-7b-3p had significant differences, and gene enrichment analysis showed that these miRNAs and target genes were mainly involved in the occurrence of AF through affecting biological processes such as energy metabolism, lipid metabolism, inflammation and enzyme activity (107). Similar studies have found that miR-483-5p, miR-142-5p and miR-223-3p are also involved in the occurrence and development of AF (108). Joung et al. found that exosomes in the peripheral blood of patients with atrial fibrillation can reduce cardiomyocyte viability, lead to abnormal Ca^2+^ channel and induce reactive oxygen species (ROS) production. High-throughput sequencing found that miR-30a-5p expression was decreased in peripheral blood exosomes of patients with AF, and exosomes with high expression of miR-30a-5p could attenuate pacemaker induced Ca^2+^ channel abnormalities (109). Hou et al. screened the differential miRNAs of peripheral blood and exosomes in 40 patients with AF, and found that miR-124-3p was significantly up-regulated, and the high expression of miR-124-3p could improve the viability and proliferation ability of myocardial fibroblasts. Mechanism studies have shown that miR-124-3p can promote the activation and proliferation of fibroblasts through AXIN1 by regulating the WNT/β-catenin signaling pathway (110). Similarly, Exosomal lncRNAs are also potential biomarkers for AF. Joung et al. identified 26 differentially expressed lncrnas in serum exosomes from patients with persistent AF. lncRNAs LOC105377989 and LOC107986997 continued to increase, has significant diagnostic effectiveness for AF, and is a potential biomarker for the diagnosis of AF (106). Lei et al. using GEO database, LINC00636 was found to be an antifibrotic molecule with decreased expression in peripheral blood exosomes of patients with AF. Mechanism studies have shown that LINC00636 can promote the expression of miR-450a-2-3p, thereby inhibiting the expression of MAPK1, and thereby improve cardiac fibrosis in patients with AF (111).

## 9. Conclusions

In recent years, with the deepening of research, ncRNAs play an important role in the occurrence and development of AF. Differential expression of ncRNAs in peripheral blood of patients with AF provides a new theoretical basis for auxiliary diagnosis of AF. At the same time, ncRNAs are involved in myocardial cell remodeling and ion channel remodeling, providing a new scheme for the treatment of AF.

This manuscript reviews the research progress of ncRNAs in the occurrence, treatment and potential biomarkers of AF. According to the existing studies, we can find that ncRNAs are closely related to AF and involved in the occurrence and progression of AF, which is worthy of further study and has great clinical significance.
